# TILs in Breast Cancer: Paving New Ways Beyond Their Prognostic and Predictive Values

**DOI:** 10.3390/cancers18193085

**Published:** 2026-09-23

**Authors:** Jean-Marc Ferrero, Loïc Trapani, Rym Bouriga, Baharia Mograbi, Jocelyn Gal, Gérard Milano

**Affiliations:** 1 Department of Medical Oncology, Antoine Lacassagne Center, University Côte d’Azur, 33 Avenue de Valombrose, 06189 Nice, France; jean-marc.ferrero@nice.unicancer.fr; 2 Department of Cytopathology and Anatomopathology, Antoine Lacassagne Center, University Côte d’Azur, 33 Avenue de Valombrose, 06189 Nice, France; 3 Department of Medical Oncology, Park Imperial Hospital, 28 Boulevard du Tzarewitch, 06000 Nice, France; 4 Institute for Research on Ageing and Cancer, Nice (IRCAN), Fédérations Hospitalo-Universitaires (FHU) OncoAge, Centre National de la Recherche Scientifique (CNRS) 7284, Institut National de la Santé et de la Recherche Médicale (INSERM) U1081, University Côte d’Azur, 28 Avenue Valombrose, 06107 Nice, France; 5 Institut Hospitalo-Universitaire (IHU) RespirERA, University Côte d’Azur, 27 Rue du Professeur Delvalle, 06000 Nice, France; 6 Antoine Lacassagne Center, University Côte d’Azur, 33 Avenue de Valombrose, 06189 Nice, France

**Keywords:** breast cancer, tumor-infiltrating lymphocytes, prognostic markers, predictive markers, artificial intelligence

## Abstract

Tumor-infiltrating lymphocytes (TILs) measurement has emerged as a critical biomarker in breast cancer management, being considered as having strong prognostic and predictive values, particularly in triple-negative cases where the clinical interest of TILs determination is clearly established. The central aim of this viewpoint article is to delineate interesting new avenues for enlarging TILs’ clinical usefulness in breast cancer. First, the advent of computational methods, including artificial intelligence (AI), represents a major shift toward improving TILs assessment, potentially reducing inter-observer variability and enabling reproducible scoring across institutions. Secondly, integrating emerging immunological tumoral markers like TLS (tertiary lymphoid structures) and CRATERs (CD8+ T cell structures) with TILs could further improve treatment outcome prediction. Thirdly, TIL levels may be modulated through the optimized use of chemotherapy, anti-angiogenic agents, and cancer vaccines. More knowledge continues to be gained regarding factors influencing TIL levels, including parity and breastfeeding, exposure to infection, metabolic stress, and environmental factors that contribute to immune fitness across the lifespan.

## Simple Summary

Assuming the prognostic and predictive roles of tumor-infiltrating lymphocytes (TILs) in breast cancer management the purpose of this viewpoint was mainly to achieve three objectives. The first was to consider AI for improving TILs measurement and to reduce scoring variability. The second was to propose the integration of immunological tumoral markers like tertiary lymphoid structures in order to combine with TILs to improve treatment outcome prediction. The last objective was to envision a possible modulation of TIL levels through the application of chemotherapy, anti-angiogenic agents and cancer vaccines.

## 1. Introduction

Breast cancer represents worldwide the second most frequent tumoral localization (11.6% of all cancer globally) following lung cancer (12.4%) [1]. Breast cancer is among the most extensively studied tumor sites regarding the application of tumor markers with prognostic and predictive values [2]. This continuous production of breast cancer tumor markers began with establishing the clinical value of markers with a predictive value, like estrogen receptor (ER) expression and Human Epidermal Growth factor receptor 2 (HER2) [3], and has progressed to the more recently explored and clinically validated genomic prognostic markers [4]. These tumor markers include multigene signatures such as the well-explored 21-gene recurrence score [5], other multifactorial genomic applications [6], and a particularly promising whole-genome sequencing approach, as recently reported [7].

Tumor-infiltrating lymphocytes (TILs) may exert variable influence on patient prognosis as shown across numerous cancer types including in particular two major cancers which are colorectal cancer [8] and lung cancer [9]. TILs can be classified as intratumoral (iTILs) and stromal (sTILs). sTILs are spread in the stroma and do not have a direct contact with cancer cells while iTILs are less abundant and hard to identify. This means that almost all studies determine TILs as TILs being evaluated using H&E tumor sections [10]. For breast cancer specifically, the clinical value of TILs includes a prognostic value with tumors of high TILs content being less life-threatening due to the potential antitumor immunoreactivity, this being mainly observed in triple-negative (TNBC) and HER2-positive cases, as well as a predictive capacity as particularly well illustrated in the context of immunotherapy with immune checkpoint inhibitors [11]. Thus, the integration of TILs assessment in a routine practice represents a promising option in breast cancer management.

The present paper does not constitute a supplementary general overview of TILs in breast cancer. The aim of this position paper is first to provide a scoping overview summarizing the current status of TILs in breast cancer and secondly to propose interesting new avenues to explore for enlarging the clinical usefulness of TILs in breast cancer. The prognostic and predictive value of TILs can be interpreted in terms of the immunoprotective role of the tumor immune infiltrate, which may confer an advantage by limiting adverse tumor progression (prognostic value) and by increasing the likelihood of an effective immune response against tumor cells (predictive value for checkpoint inhibitor efficacy). Accordingly, this paper will consider several key aspects, including criteria for TILs tumor characterization and the current strategies for modulating intratumoral TIL levels. The paper will also explore new perspectives on TILs when combined with other tumor-associated immune structures, such as tertiary lymphoid structures [12] and the recently identified CRATERS [13].

The current article is based on a comprehensive literature review of TILs in breast cancer, incorporating reviews, meta-analyses, and both prospective and retrospective studies. Emphasis was placed on large patient cohorts and the most recent research in this field (approximately 70% of the quoted references were within the last 3 years). We apologize to authors whose important contributions may not have been cited, since this work was designed as a scoping review rather than as an exhaustive overview.

## 2. Established Status of TILs in Breast Cancer

### 2.1. Prognostic Value of TILs

TNBC constitutes 10 to 15% of breast cancer patients [14]. This category of patients is defined by the absence of ER and HER2 expression, and its prognosis is particularly unfavorable; tumor size and node involvement remain the only clinically relevant prognostic indicators [15]. Independent of the application of adjuvant chemotherapy, the quantity of TILs is associated with disease-free survival as well as overall survival in TNBC patients [16]. In a large series of 476 TNBC patients representing a population coming from four independent cohorts, an average TIL level of 10% was reported in the tumors examined [17]. Among this group of patients, those categorized as stage I with TILs greater than 30% had a 5-year disease-free survival of 91%.

A controlled French multi-institutional prospective study of TNBC patients without node involvement (T1b, T1C) is currently underway [18]. This trial aims to apply an adjuvant treatment based on TIL counts. Thus, patients with TILs greater than 30% will receive adjuvant chemotherapy with a taxane–pembrolizumab combination. A long-term follow-up of these patients is planned to appreciate both the prognostic and predictive value of TILs in TNBC. More generally, high levels of TILs were found to be associated with improved clinical outcomes, particularly in aggressive breast cancer subtypes like TNBC, as well as HER-2 positive cases [19,20].

### 2.2. Predictive Value of TILs

The presence of TILs may also be of predictive value in TNBC patients, and a high quantity of TILs has been shown to correlate with a higher probability of pathologic complete response following adjuvant therapy for these patients [21]. Of note, pivotal immunotherapy trials have been particularly revealing in the predictive value of TILs in breast cancer. In early TNBC, when pembrolizumab is added to neoadjuvant chemotherapy in the phase Ib KEYNOTE-173 trial, it significantly increases pathologic complete response (pCR) rates (*p* = 0.0085) for patients with a high baseline stromal TILs (sTILs) [22]. PCR rates reached nearly 74% for patients with sTILs ≥ 30% in the NeoPACT-a phase II trial, using an anthracycline-sparing regimen of pembrolizumab + carboplatin (AUC 6) + docetaxel. In both, free survival was higher in patients whose tumors had high TILs and high PD-L1 expression, a pattern observed in early TNBC from the phase III KEYNOTE-522 study [23,24]. Of note, pathological complete response rates exceeded 80% in real-world data post-KEYNOTE-522 for high-TILs TNBC treated with pembrolizumab plus chemotherapy [25]. In metastatic TNBC, when TILs were elevated, objective response rate, progression-free survival, and OS increased with single-agent pembrolizumab, as shown in the phase III KEYNOTE-119 trial [26]. Li and coworkers reported a meta-analysis of studies on TILs in breast cancer patients treated with neoadjuvant chemotherapy (NACT) [20]. Among 29 eligible studies, the authors concluded that increased TIL levels could significantly predict response to NACT in HER2-positive and triple-negative patients, but not in ER-positive patients. The authors attributed these differences in the predictive value of TILs to the nature of the tumor-infiltrating immune cells, noting that B cells and myeloid dendritic cells are associated with improved outcomes in ER-positive tumors [20].

Following these observations, studies applying pre-treatment TILs as a stratification marker are increasingly being used to guide treatment selection or de-escalation. It was already noted that the phase II ETNA trial (NCT06078384) enrolls early TNBC patients with sTILs ≥ 30% to evaluate the omission of chemotherapy and to randomize them to pembrolizumab versus observation [18]. Such a TILs cutoff, however, is not generalizable, and a French multicenter phase III investigator-initiated protocol (ETNA trial, NCT01822314) integrates TILs cutoff criteria into eligibility or stratification models to enable chemotherapy de-intensification in immune-hot early TNBC and HER2-positive disease. A-BRAVE is a phase III trial that explored the importance of timing of immunotherapy delivery in breast cancer in an adjuvant setting [27]. A baseline TILs level was found to discriminate the importance of immunotherapy timing. Thus, for patients with a baseline TILs below 20%, the PM-dominant administrations led to a higher survival than the AM-dominant ones. No such difference was observed for TILS above 20%.

### 2.3. TILs and Practical Implications in Breast Cancer

TILs measurement has practical implications for clinical decision-making, as summarized in Table 1 across different breast cancer subtypes. Table 1 indicates that TN breast cancers benefit the most from TIL measurements in both prognostic and predictive value. But other subtypes, like HER2-positive and, to a lesser degree, luminal (ER+/HER2−), can also gain from TILs determination. Notably, TIL levels vary markedly across tumor subtypes (Table 1). Above all, the accumulated knowledge points to a clinical relevance of TILs in breast cancer, which still depends on the subtype.

Of practical importance, baseline TILs may also serve as a continuous predictor, with higher values correlating with better outcomes, and composite TILs-PD-L1 scores predict immunotherapy response in TNBC with up to 80% specificity across subtypes [11]. It was also shown that for HER-positive breast cancer patients included in a well-designed controlled trial [33], a TILs value of 20% or higher allowed treatment de-escalation without risking distant metastasis or death.

## 3. New Ways to Pave with TILs

### 3.1. Tumor TILs Characterization

TILs are intricately embedded within the tumor itself and the tumor microenvironment (TME), which consists of a diverse array of immune cell types, including CD8+ TILs, regulatory CD4+ T cells, NK cells, macrophages, T helper cells, and myeloid-derived suppressor cells [34]. More precisely, in TNBC, various TIL subtypes have been identified, each with unique cell-surface markers and differing prognostic significance [35]. Therefore, accurately detecting and quantifying TILs presents a significant challenge for investigators seeking to validate TILs scoring in clinical practice [36,37].

Because TILs are present in both tumor tissue and stroma, international working groups recommend measuring TILs primarily in the stroma to enhance reproducibility [38]. Specifically, it is advisable to avoid ischemic and necrotic areas, as well as regions enriched with polymorphonuclear neutrophils [39]. Recently, Baharum and colleagues reported that heterogeneous TILs distribution, unclear tumor–stroma interfaces, and dense focal lymphoid infiltrates are key factors leading to considerable variability in TILs measurement [40]. More generally, conventional manual assessment of TILs comes with certain limitations, such as inter-reader variability and subjectivity, which can introduce a non-negligible risk of bias [41]. However, concordance studies among pathologists measuring TILs generally show satisfactory results when standard recommendations are strictly followed [41]. Key issues remain inherent to TILs evaluation, covering reproducibility, inter-laboratory variability, and a lack of standardized scoring.

To address these challenges, there has been a welcome emergence of automated TIL scoring methods that employ promising deep learning techniques. A recent systematic review focused on three keywords (breast cancer, artificial intelligence, and TILs) to evaluate the performance of these automated TILs scoring methods in breast cancer [36]. The authors found that semantic segmentation, object detection, and convolutional neural networks were the most commonly used methods for automated TILs scoring. Moving forward, standardizing algorithms and conducting clinical validations constitute the essential next steps in this promising field of TILs quantification, especially regarding breast cancer. For instance, the ECTIL model (label-efficient computational stromal TILs assessment) was applied to a cohort of 2340 breast cancer patients, including 790 with TNBC [42]. Remarkably, without using deep learning-based segmentation, ECTIL produced hazard ratios comparable to those from pathologists in overall survival analyses, independent of traditional clinicopathological variables.

### 3.2. Intratumoral TILs Modulation

Given the potential clinical importance of TILs in breast cancer, the next question may ask how to modulate the tissue content of T lymphocytes. With this in mind, one can consider, on the one hand, modulation of natural origin that confers biological effects on the immune system, and, on the other hand, changes in T cell infiltration in the tumoral tissue due to cancer treatments.

Different approaches are offered for this latter consideration as illustrated in Figure 1. First, there is a potential role for chemotherapy, with mechanistic evidence recently reported from the production of CA-rich DNA fragments in breast cancer tissue, which has the capacity to increase tumor antigen-reactive T cells [43]. Surprisingly, a systematic review and meta-analysis revealed that, based on a series of 2072 patients, there was a significant decrease in TILs content following neoadjuvant chemotherapy in breast cancer [44]. The authors argued that as the tumor shrinks under chemotherapy, danger signals decrease, and the antitumor immune response may be weakened as a result. Clearly, more experimental and clinical studies are needed to examine the evolution of TILs in breast cancer tissue during and after chemotherapy, before claiming a definitive role of this treatment modality in TILs modulation. It will also be interesting to determine the role of the chemotherapy administration modality itself in designing optimal combinations of chemotherapy and immunotherapy, given the potential immunoreactivity against tumor cells attributable to intratumoral T cells. For example, following continuous low-dose chemotherapy (metronomic chemotherapy), the tumor microenvironment might shift from an immunosuppressive to an immunostimulatory state due to vascular normalization, restoring an optimal intratumoral ratio of Tregs and CD8+ T cells [45].

In fact, tumor-associated vasculature (TAV) represents a true opportunity to overcome the limitation of T cell homing [46]. As a representative example of immunomodulation via targeting of the tumoral vasculature, anti-VEGFR2 treatment in mouse breast cancer models has been shown to promote TAV normalization and immune cell infiltration [47].

The underlying molecular mechanisms of the crosstalk between angiogenesis and tumor immunity are numerous and have recently been reviewed [48]. Interestingly, the authors noted that following anti-angiogenic treatment, nitric oxide inhibition increased the sensitivity of anergic endothelial cells to inflammatory cytokines, promoting T cell adhesion to the endothelium and subsequent transmigration.

One of the main objectives of cancer vaccination is to induce or enhance T cell reactivity against tumor cells that express identifiable antigens. It is well established that therapeutic vaccines represent a logical and promising strategy for reversing a T cell-deprived TME [49]. Breast cancer vaccines can now be protein-based or involve whole cells, mRNA, or dendritic cells [50]. Notably, a new vaccine platform has been reported that enables a multifunctional mRNA/siRNA nanovaccine for TNBC treatment [51]. The platform was used to deliver *MUC1* mRNA and *CTLA-4*si RNA and was shown to enhance TILs in tumors. These data were obtained in an animal model; accordingly, clinical confirmation is eagerly awaited, as this platform shows strong potential for clinical use.

On the natural side of TILs modulation, there is well-established evidence that the immune system undergoes age-related changes, particularly affecting cell composition, function, and regenerative capacity. There is emerging and promising research at the crossroad between aging, immune fitness, and cancer [52]. In this context, Takada and colleagues retrospectively examined TILs density in 356 breast cancer patients and found that older patients with TNBC had significantly lower TILs infiltration than younger patients. In line with this observation, younger TNBC patients (below 45 vs. above 60) had significantly longer survival [53]. These differences could lead to a better selection of treatment modalities based on the immunogenicity of breast cancer subtypes. More knowledge remains to be gained, however, about the cumulative impact of mixed and variable exposures to infection, metabolic stress, and environmental factors on immune fitness across the lifespan [52].

It is well known that parity and breastfeeding may reduce the risk of breast cancer onset, but the biological mechanisms underlying this protection were not well established until now. More precisely, Virassamy and colleagues recently reported the interesting finding that parity can lead to an accumulation of CD8+ T cells in normal human tissue. These human data were supported by an experimental study in a murine model showing that pregnancy followed by lactation led to an accumulation of CD8+ T cells in the mammary gland, consistent with reduced growth of grafted breast tumor models, and an increase in intratumoral immune cells [54]. Importantly, the authors noted that TNBC from parous women were characterized by greater T cell infiltration and a more favorable clinical outcome. Notably, the T cell infiltrates persisted for over 30 years. Thus, among other points, this work could position CD8+ T cells as a major contributor to parity-associated protection, which can also favorably influence the outcome of further-developed breast cancers. It follows that capturing reproductive history along with the information about TILs content could be of clinical importance for optimal management of breast cancer.

### 3.3. Perspectives

Several promising perspectives are emerging for TILs assessment in breast cancer. For instance, beyond conventional TILs, expanding analysis to include CD8+ T cell and plasma cell densities in both the tumor core and infiltrative margin may enhance prognostic accuracy in TNBC [55]. However, this approach requires further clinical validation. TILs, along with Ki-67 and PD-L1 expressions, are among the most commonly reported markers predicting response to neoadjuvant chemotherapy in TNBC [56]. A combined analysis of these biological parameters would be justified at least in TNBC. Additionally, artificial intelligence (AI) and digital pathology are advancing rapidly [57] and offer promising tools for automated TILs assessment, particularly for analyzing spatial immune–tumor interactions associated with clinical outcomes. Recent studies have demonstrated that AI-derived TIL metrics can be prognostic across different cancer types and predictive of immunotherapy response, supporting their potential clinical utility [58,59]. However, many models lack robust external validation and remain sensitive to variability in staining, slide preparation, and scanner technologies, leading to domain shift and reduced generalizability in real-world settings. Furthermore, annotation bias, inter-observer variability, and technical challenges in accurately distinguishing tumor, stroma, and inflammatory compartments may limit reliability. As highlighted in recent breast cancer-focused analyses, the field is moving from proof of concept to implementation, where robustness and reproducibility are critical [57,60]. While bulk and single-cell RNA sequencing provide higher-resolution immune profiling, their cost and scalability currently restrict routine use [61], reinforcing the appeal of AI-based approaches, provided that standardization and multicenter validation are achieved.

Interestingly, emerging biomarkers may, along with TILs, offer additional predictive value. Tertiary lymphoid structures (TLS) organized as lymph node-like aggregates, linked to CD8+ T cell infiltration, optimize antigen presentation, T cell expansion, and activation [12]. Mattiuz and coworkers reported a pivotal role of mature dendritic cells in both establishing and organizing TLS functionality [62]. The authors found that dendritic cells favorized MHC-II and MHC-I antigen presentation to TILs within tumor lesions allowing TLS to become functional. Interestingly, the presence of TLS correlated with improved overall survival in NSCLC [62]. Similarly, the presence of additional structures called CRATERs (CD8+ T cell structures) that breach the stroma into the tumor predicts clinical benefit in patients receiving immunotherapy [13]. Thus, integrating TLS and CRATER assessment with conventional TILs could substantially improve treatment outcome prediction, particularly for breast cancer immunotherapy.

## 4. Conclusions

There is good evidence for the clinical utility of TILs measurement in breast cancer, this being based on well-established prognostic and predictive values, particularly in TNBC. However, TILs determination in breast cancer must move from a powerful diagnostic tool to a true driver of clinical decision making. Thus, establishing TILs’ clinical utility rather than bringing further prognostic validation should now be the priority. Conventional TILs assessment suffers significant limitations, including variable antibody and assay sensitivities, that complicate the establishment of standardized TIL thresholds for clinical decision-making. The existence of different positivity cutoffs, assays, and platforms remains a critical barrier to implementing TILs as a reliable and generalizable companion diagnostic biomarker for breast cancer management. Encouragingly, computational assessment is driving substantial progress that may reduce inter-observer variability and improve the reproducibility of faithful scoring. Three main challenges remain for TILs measurability with a large-scale validation of AI-assisted TILs measurement, with the demonstration of inter-cohort robustness, and with enhanced interpretability. Successfully addressing these challenges will establish TILs measurement as a faithful companion biomarker for breast cancer management, particularly for immunotherapy selection and evidence-based treatment de-escalation strategies. Additional areas of clinical interest also warrant further investigation, including HER2-low cases [63] and patients with early-stage breast cancer [64]. Above all, efforts must be dedicated to the obtention of prospective evidence showing that TILs-guided treatment can improve patient outcomes.

## Figures and Tables

**Figure 1:**
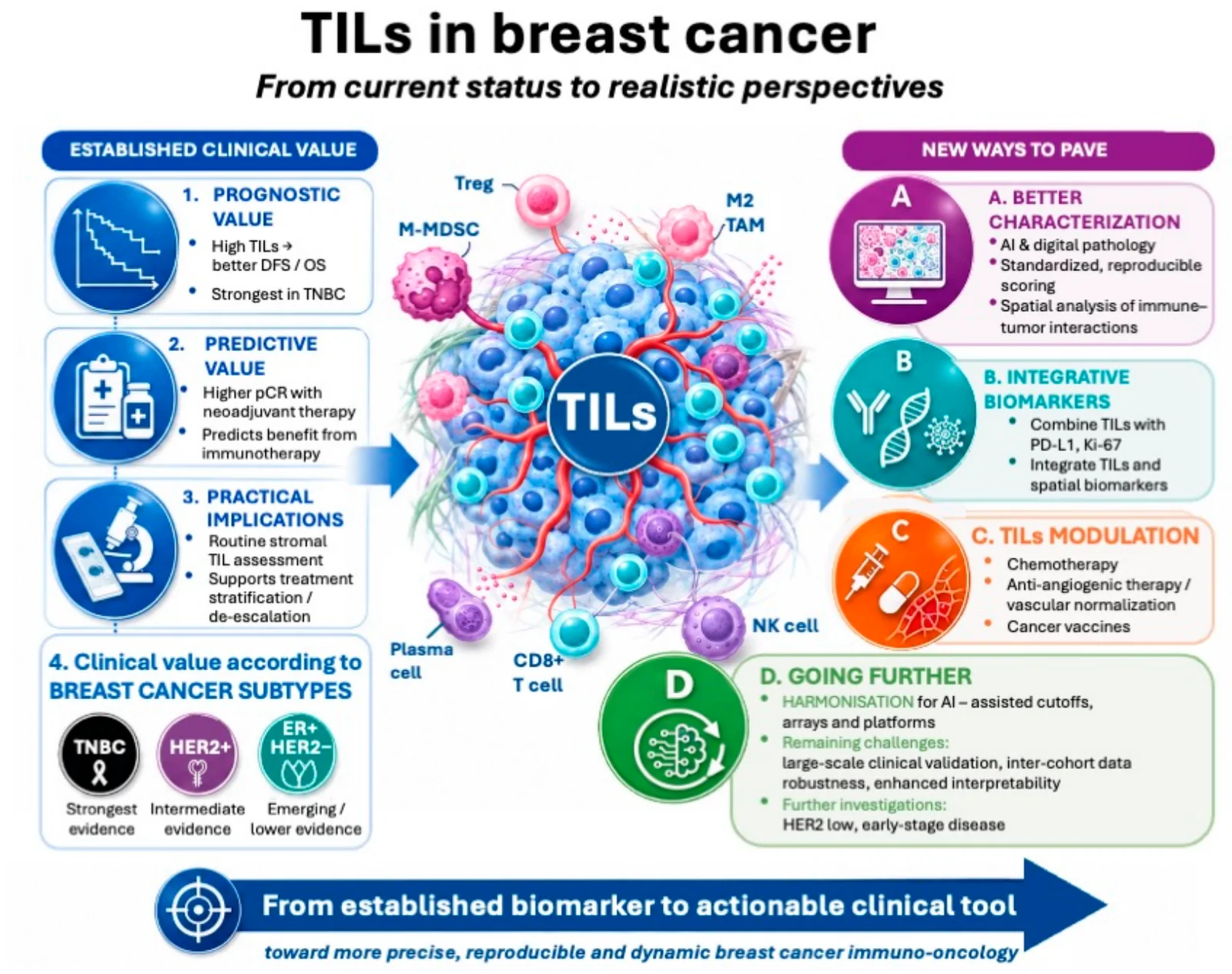
An illustrative abstract depicting the evolution with landscape of TILs in breast cancer from an established clinical value towards new ways to pave and remaining challenges. Created and developed with assistance from ChatGPT (OpenAI, San Francisco, CA, USA).

**Table 1:** A synthetic view on TIL prognostic and predictive values in breast cancer.

SUBTYPE	TILs Average Levels [28]	Level of Evidence	Prognostic Value	Predictive Value
**Triple negative (TNBC)**	Highest among all subtypes	High	Strong prognostic value: higher TILs associated with improved survival and pCR [11,19,20,29]	Predictive of response to neoadjuvant chemotherapy and checkpoint inhibitor therapy [11,22,29]
**HER2 positive**	Intermediate levels	Moderate	Associated with improved survival outcomes in long-term follow-up [10,30]	Predictive of response to neoadjuvant therapy, especially trastuzumab-containing regimens [10,22]
**ER+/HER2−**	Lowest among all subtypes	Low to Moderate	Limited prognostic utility; modest association with outcomes in some subsets [11,31]	Higher stromal TILs linked to pCR in high-grade ER+/HER2− tumors [31]; weak predictive signal overall [32]

**Legend—Level of Evidence definitions: High evidence**: Multiple large-scale studies, meta-analyses, or pooled analyses (e.g., ≥1000 patients) with consistent results across independent cohorts and study designs. Includes randomized trial data or prospective validation [21,22,29]. **Moderate evidence**: Single randomized trial analyses, moderate-sized cohort studies, or meta-analyses with some heterogeneity across included studies. Findings generally consistent but with subtype-specific variability [10,32]. **Low evidence**: Exploratory or retrospective analyses, small cohorts, or studies with conflicting results. Evidence suggestive but not conclusive; further validation needed [31].

## Data Availability

No new data were created or analyzed in this study. Data sharing is not applicable to this article.
